# SNP-RFLPing: restriction enzyme mining for SNPs in genomes

**DOI:** 10.1186/1471-2164-7-30

**Published:** 2006-02-17

**Authors:** Hsueh-Wei Chang, Cheng-Hong Yang, Phei-Lang Chang, Yu-Huei Cheng, Li-Yeh Chuang

**Affiliations:** 1 Faculty of Biomedical Science and Environmental Biology, Kaohsiung Medical University, Taiwan; 2 Department of Electronic Engineering, National Kaohsiung Univeristy of Applied Sciences, Taiwan; 3 Chang Gung Bioinformatics Center, Taiwan; 4 Department of Chemical Engineering, I-Shou Univeristy, Taiwan

## Abstract

**Background:**

The restriction fragment length polymorphism (RFLP) is a common laboratory method for the genotyping of single nucleotide polymorphisms (SNPs). Here, we describe a web-based software, named SNP-RFLPing, which provides the restriction enzyme for RFLP assays on a batch of SNPs and genes from the human, rat, and mouse genomes.

**Results:**

Three user-friendly inputs are included: 1) NCBI dbSNP "rs" or "ss" IDs; 2) NCBI Entrez gene ID and HUGO gene name; 3) any formats of SNP-in-sequence, are allowed to perform the SNP-RFLPing assay. These inputs are auto-programmed to SNP-containing sequences and their complementary sequences for the selection of restriction enzymes. All SNPs with available RFLP restriction enzymes of each input genes are provided even if many SNPs exist. The SNP-RFLPing analysis provides the SNP contig position, heterozygosity, function, protein residue, and amino acid position for cSNPs, as well as commercial and non-commercial restriction enzymes.

**Conclusion:**

This web-based software solves the input format problems in similar softwares and greatly simplifies the procedure for providing the RFLP enzyme. Mixed free forms of input data are friendly to users who perform the SNP-RFLPing assay. SNP-RFLPing offers a time-saving application for association studies in personalized medicine and is freely available at .

## Background

SNP genotyping is essential for association studies in personalized medicine. Although many high-throughput SNP genotyping methods have been reported, lots of researchers still report their SNP genotyping by restriction fragment length polymorphism (RFLP). NEBcutter [1] can provide the RFLP information for any input sequences using REBASE information [2]. However, it is not convenient for SNP related sequences. To discriminate one SNP in a RFLP assay, the restriction enzymes have to recognize only one of the SNP containing sequences. Therefore, the users have to input data twice for each SNP related sequence when checking for the available restriction enzymes. On the dbSNP of NCBI [3], each SNP is named in reference cluster IDs (rs) and in NCBI assay IDs (ss). Users can input the SNP ID, gene name (HUGO) or gene ID for Entrez gene in NCBI to get the SNP with its flanking sequences using NEBcutter [1]. However, it is time consuming if a gene like TP53 contains hundreds of SNPs.

In this paper, we present the web-based integrated system called SNP-RFLPing for SNP ID information and its availability for restriction enzymes. Users can input any formats of SNPs including NCBI dbSNP rs or ss ID, HUGO gene name and gene ID for Entrez gene in NCBI [4]. Then, the availability of restriction enzymes as well as SNP-related information can be presented. It also functions for user-defined SNPs, which are not reported in the NCBI database. For large data of SNP IDs or gene IDs, SNP-RFLPing provides a file upload service to perform the RFLP assay for efficient screening of SNP-RFLP enzymes in association studies.

## Implementation

**Figure 1:**
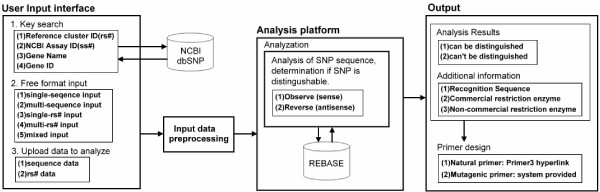
SNP-RFLPing web-based flowchart. Three kinds of functions are incorporated in the SNP-RFLPing system, namely a user input interface, an analysis platform, and an output module. The user input interface contains 1.) Key search, 2.) Free format input, and 3.) Upload data for analysis. Only key search is mining from NCBI dbSNP [3]. The other interfaces of the input interface are programmed to input data preprocessing, and then transfer the data to the analysis platform. In the analysis platform, SNP-containing sequences are transferred to a local database downloaded from REBASE [2] and then the RFLP availability for the sense and antisense sequences are analyzed. Finally, the result is transferred to the output module. Under output, SNP-RFLPing availability is provided, as well as other SNP information and primer design.

SNP-RFLPing, a web-based interface, was designed and implemented under the SQL server database system. Java server pages and Java applets are used to input data and file processing between the users and the applications, and to parse the data, respectively. The workflow of SNP-RFLPing is illustrated in Figure 1. We found that the KMP algorithm [5] tested takes a long time due to the human SNP database's huge size. To improve the matching efficiency, the Boyer-Moore algorithm [6] was chosen in this system and performed well. Database structure is mainly set up by REBASE [2] and NCBI dbSNP [3], which are transformed into the MySOL format and a local copy database, respectively.

## Results

### Input data

**Figure 2:**
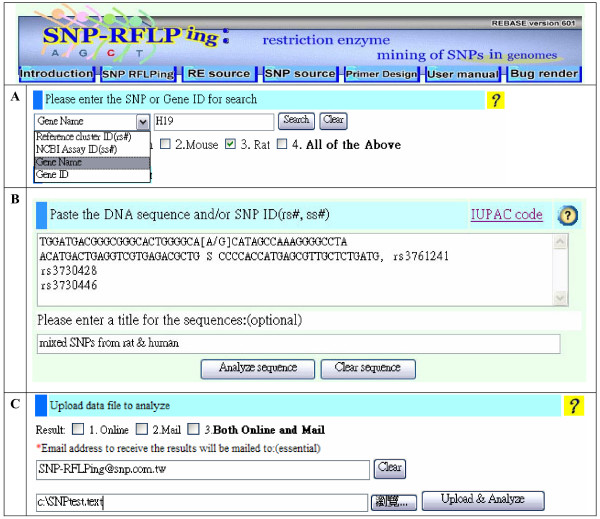
Input items for RFLP availability and SNP related information. (A) SNP ID in rs# and ss# formats and gene in HUGO and ID formats are acceptable for SNP-RFLPing assay. Human, mouse and rat genomes are included. (B) Freely mixed forms of multiple inputs, including sequences and SNP ID with rs# are acceptable for SNP-RFLPing assay. Different events of input sequences and/or SNP ID are separated by the comma symbol "," or by different lines using the "Enter" key on the computer keyboard. IUPAC format or [dNTP1/dNTP2] format are acceptable in this software. Empty spaces while inputting data doesn't interfere with the screening. The IUPAC code is provided online. (C) All the results can be displayed online, by email or both.

**Figure 3:**
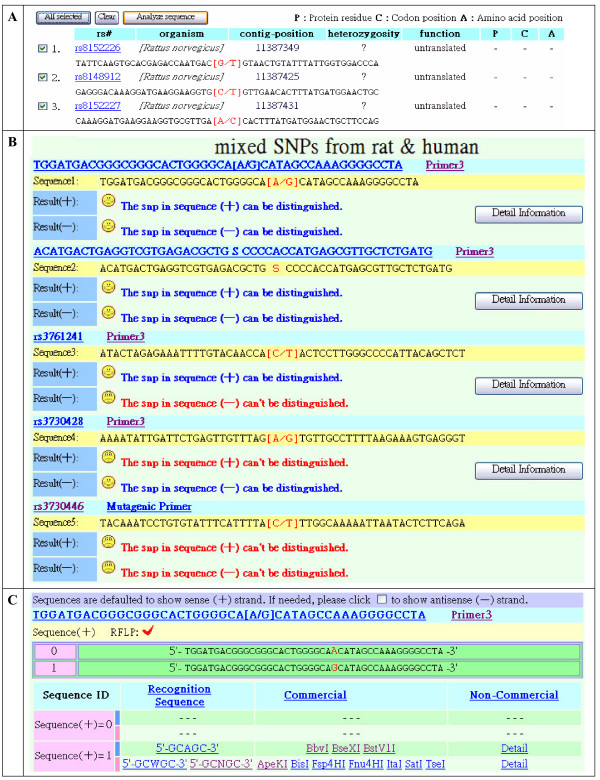
Output items for RFLP availability and SNP related information. (A) Results of input for the gene name of HUGO. H19 (gene input in Figure 2A) is used as example. Here, only part of the SNP information is shown. The SNP ID in rs#, organism, contig-position, heterozygosity, function, protein residue, codon position, and amino acid position are shown. Each SNP in the SNP list for the input gene is shown in the order of its contig position. The system provides the partial or entire selections for SNPs. (B) Detailed information results are shown by inputting information from Figure 2B. SNP information from different species is acceptable. Each SNP-containing sequence was automatically transformed into sense and antisense strands marked with "+" and "-", respectively. After selection by the first sequence with SNP (= rs8144801), the results are shown in Figure 3C. (C) Standard results of SNP-RFLPing demonstrate detailed information of restriction enzymes and their target site in each strand if available. The system shows only the "+" (sense) strand by default. The hind "-" (antisense) strand can be shown by selection a checkbox. The alternative SNP-containing sequence is separated into two sequences marked with "0" and "1". When the sequences are suitable for restriction enzymes, the RFLP result shows "V". In contrast, if no RFLP is available in the restriction enzyme "X" is shown. Both commercial and non-commercial restriction enzymes are divided into two parts marked with blue and red colors to represent the recognition site for endonucleases with and without degenerated nucleotides, respectively.

Inputs of SNP-RFLPing are line fed through its web interface for the human, rat or mouse SNP-RFLP assay. The gene name (HUGO), gene ID (Entrez gene in NCBI), and SNP ID (rs#, ss#) from these species are accepted formats for SNP-RFLPing (Figure 2A). To provide users-friendly formats, this software was designed to accept the mixed inputs of the sequences with NCBI or user-defined SNP formats (IUPAC or dNTP1/dNTP2), as well as SNP ID rs# or ss# at the same time (Figure 2B). "A", "T", "G", "C" are accepted as they are. Other ambiguous letters are regarded according to the IUPAC system. Upper and lower case is not significant and all other characters, including spaces and digits, are ignored. Batch input is available for screening at the same time for line feeds or using the comma "," on the computer keyboard. Data upload, online output, as well as email output are supported (Figure 2C). The output results for the sequence in Figure 2A and 2B are shown in Figure 3A and 3B, respectively.

### Output data

SNP related information is provided for the RFLP assay including the SNP ID, species, contig-position, heterozygosity, function, protein residue (P), codon position (C), and amino acid position (A) (Figure 3A). It may be helpful for the users to select interesting SNP targets for association studies. The analyzed SNP can be selected as a whole or partially at the square box. Then, the RFLP availability of the restriction enzymes for the input SNP-containing sequence (marked as +) and its complementary sequence (marked as -) is shown separately in Figure 3B. The commercial and non-commercial restriction enzymes shown in Figure 3C are linked to restriction enzyme databases REBASE [2]. SNP-RFLPing provides a mutagenic (or mismatched) primer for a SNP in which a suitable restriction enzyme can not be found naturally. The optimal primer design follows criteria as described [7,8], such as melting temperature, length, and base composition. The primer opposing to the mutagenic primer and the natural primer sets can be designed using Primer3 [9], which is hyperlinked in the software.

## Discussion

In this paper, we propose a web-based interface and a java-based program, SNP-RFLPing, to provide SNP ID-based (rs# and ss#), gene-based (gene name and ID) and SNP-in-sequence-based RFLP analysis from the REBASE and dbSNP database. In Table 1, feature comparisons are made between SNP-RFLPing and other existing RFLP assay tools, including: SNPicker [10], NEBcutter [1], SRP Opt [11], PIRA-PCR Designer [12], SNP cutter [13], SNPselector [14], SNP2CAPS [15], and software from the in-silico company [16]. The results indicate that SNP-RFLPing is more efficient and informative than other tools, especially with regards to input data preparation, free sequence input format requirement, gene-based SNP-RFLP assay, and detailed output content for SNP information (Table 1). For example, some of programs allow only sequence input and limit their application, e.g., SNPicker, NEBcutter, SRP Opt, PIRA-PCR Designer, and SNP2CAP. Only SNP-RFLPing, SNP cutter [13] and software from the in silico company [16] provide the input of SNP ID and sequences to screen RFLP information. However, the SNP data in SNP cutter needed the specified input of SNP-in-sequence, i.e., (gene name) (SNP1_SNP2) (5'- flanking and 3'-flanking). In contrast, SNP-RFLPing accepts any common formats to check for RFLP availability for a SNP with its flanking sequence. IUPAC and [dNTP/dNTP] formats are both allowed in the SNP sequences, as shown in Figure 2B. While the software from the in silico company [16] also allows multiple pre-aligned sequence formats when comparing multiple SNPs simultaneously. The length of cutting fragment using restriction enzyme is also provided.

In the SNP-RFLPing server, more input items are provided, including: rs#, ss#, gene name, and ID for human, mouse and rat genomes. It is very convenient for a user to check the available restriction enzyme for each gene of interest, both online and per email. To our knowledge, SNP-RFLPing is the first software to link the gene name and its SNP-RFLP restriction enzyme. It's not necessary to search all SNPs of a certain gene from the NCBI dbSNP [3] before putting all these SNPs into a suitable SNP-RFLP software, like SNP cutter [13]. SNP500Cancer [17] also provides SNP searching by genes, but doesn't provide the RFLP function, and the coverage of SNPs is limited to human cancer-related genes. In SNP-RFLPing, only one step is needed without transforming specific formats before assay. This design will speed up the screening with SNP-RFLPing compared to other available software.

In addition to RFLP enzymes, RFLP genotyping also needs the primers for PCR-RFLP. Softwares like PIRA-PCR [12], SNP cutter [13], and software from the in-silico company [16] can provide a design function for mutagenic primers (Table 1). Similarly, SNP-RFLPing provides the newly developed mutagenic primer designer. We also provide a hyperlink to the freely available software Primer3 [9] for the design of primers opposing to mutagenic primer and the natural primer sets. The path for primer design in SNP-RFLPing will be integrated in the future. Alternatively, we recommend a user to use SNP-RFLPing software coupled with other commercial primer designers, e.g., Beacon Designer 4 (Premier Biosoft International, CA), which are usually unable to provide the RFLP information, but provide a fast and friendly natural primer design for each SNP.

## Conclusion

The web-based software, SNP-RFLPing, can solve the input format problems inherent in similar software, and greatly simplify the procedure for providing the RFLP enzyme. A novel function of SNP-RFLPing is that it can accept any common input formats to check the RFLP availability in human, mouse, and rat genomes. In addition, the searching of SNP and RFLP information by gene name is a very powerful tool for association studies with a target gene. In conclusion, it is time-saving and user-friendly to use SNP-RFLPing for association studies in personalized medicine.

## Availability and requirements

Project name: SNP-RFLPing: restriction enzyme mining in genomes.

Project home page: 

Operating system(s): Microsoft Windows XP

Programming language: Java

Other requirements: Java 1.5.0, Tomcat 5.5, SQL server 2000, MySQL 4.0

License: none for academic users.

For any restrictions regarding the use by non-academics please contact the corresponding author.

## List of abbreviations

SNP, single nucleotide polymorphism

RFLP, restriction fragment length polymorphism

NCBI, National Center for Biotechnology Information

HUGO, Human Genome Organization

REBASE, The Restriction Enzyme Database

## Authors' contributions

H-WC provided the biochemistry background, introduced the bioinformatics for SNP-RFLPing and wrote the manuscript. P-LC participated in the earlier development of the program. C-HY instructed Y-HC in writing and testing the algorithm of this software. L-YC coordinated and oversaw this study.

## Figures and Tables

**Table 1:** Comprehensive table for comparison of the features of RFLP related software.

	**Input format**	**Output format**	**Type of program**	**Flanking sequence length**	**Graphical display**	**Design of mutagenic primers**
**SNP-RFLPing**	1.rs# IDs, ss# IDs. 2.Many IDs per line (separated by comma symbol). 3.HUGO gene name. 4.Entrez Gene ID. 5.SNP-in-sequences of IUPAC or [dNTP/dNTP] format. 6.Multiple mixed forms: rs#, ss# and sequences are accepted (separated by comma or enter key).	1.On-line and/or email. 2.SNP information provided, e.g., contig position, heterozygosity, function, protein codon. 3.RFLP information, e.g., recognition site, cutting position and strand of restriction enzyme. (visualization of the result). 4.Gene-SNP-RFLP function. (Users can input the gene name and the system can provide all RFLP SNPs of the gene)	Web-Server	User input	Yes	Yes
**SNPicker **[10]	1.sequences only.	1.RFLP information, e.g., recognition site, cutting position and strand of restriction enzyme. 2.no SNP information.	Down-load	User input	Yes	Yes
**NEBcutter **[1]	1.sequences only. 2.no SNP related input.	1.on-line only. 2.no SNP information.	Web-Server	User input	Yes	No
**SRP Opt **[11]	1.sequences only. 2.microbial genome only.	1.not for SNP genotyping. 2.selection of forensic markers.	Down-load	Not mentioned	Yes	No
**PIRA-PCR Designer **[12]	1.sequences only.	1.on-line only. 2.no SNP information.	Web-Server	User input	No	Yes
**SNP cutter **[13]	1.rs# IDs. 2.one ID per line. 3.Specific sequence format (additional software needed).	1.Email only. 2.no SNP information.	Web-Server	2000 bp	No	Yes
**SNPselector **[14]	1.upload input only. 2.rs# IDs. 3.gene name. 4.genome regions. 5.no sequence input.	1.Email only. 2.no RFLP information.	Web-Server	200 bp	Yes	No
**SNP2CAPS **[15]	1.alignment sequences. 2.FASTA format only.	1.no auto-mining for RFLP enzyme.	Down-load	Not mentioned	Yes	No
**In silico software ** [16]	1.SNP ID. 2.sequences with SNP or mutation. 3.unaligned or multiple prealigned sequences are accepted.	1.on-line only. 2.SNP position in sequence. 3.RFLP information (including cutting position, selectable function for minimum recognition size, type of enzymes and commercial sources.). 4.multiple SNPs can be compared simultaneously. 5.other useful related tools provided.	Web-Server	User input	Yes	Yes
